# Self-perception and quality of life among overweight and obese rural housewives in Kelantan, Malaysia

**DOI:** 10.1186/s12955-015-0210-z

**Published:** 2015-02-12

**Authors:** Wan Abdul Manan Wan Muda, Dieudonne Kuate, Rohana Abdul Jalil, Wan Suriati Wan Nik, Siti Azima Awang

**Affiliations:** Department of Nutrition, School of Health Sciences, Health Campus, Universiti Sains Malaysia, Kubang Kerian, Kelantan 16150 Malaysia; Department of Biochemistry, Faculty of sciences University of Dschang, PO Box 67, Dschang, Cameroon; Department of Community Medicine, School of Medical Sciences, Health Campus, Universiti Sains Malaysia, 16150 Kubang Kerian, Kelantan Malaysia; Program of Dietetics, School of Health Sciences, Health Campus, Universiti Sains Malaysia, 16150 Kubang Kerian, Kelantan Malaysia; Hospital Universiti Sains Malaysia, 16150 Kubang Kerian, Kelantan Malaysia

**Keywords:** Overweight, Obesity, Quality of life, Self-perception, Rural housewives

## Abstract

**Introduction:**

Obesity, in the past was perceived to be the problem of the rich, but recent studies have reported that the problem of obesity is a worldwide problem and rural population is no less affected. Self-perceived health and weight appropriateness is an important component of weight-loss and eating behaviors and may be mediated by local, social and cultural patterning. In addition to the quality of life assessment, it should therefore be an important focal point for the design and implementation of clinical and public health policies.

**Methods:**

The present study was carried out to assess the self-perception of weight appropriateness as well as the quality of life of overweight and obese individual among the rural population particularly among housewives. A total of 421 respondents participated in the study which consisted of 36.6% in the overweight and 63.4% in the obese categories.

**Results:**

the analysis of the survey revealed that self-perception regarding obesity among respondents show common similarities, particularly in self reporting on health, dietary habit and also the concept of beauty and a beautiful body. Character and behavior are highly regarded in evaluating a person’s self-worth in society. The results on the quality of life using the ORWELL 97 instrument show that the quality of life of respondents was moderate. Most of the respondents were aware of their body weight and indicated an intention to lose weight but also reported themselves as healthy or very healthy.

**Conclusion:**

The results of the survey indicated that perception on obesity did not differed very much between respondents, in fact there existed a lot of similarities in their perception about health, quality of life, personal health and self-satisfaction with own body. However, their quality of life was within the normal or moderate level based on the ORWELL 97 assessment. Even though most of the respondents were aware of their body weight and indicated an intention to lose weight they also reported themselves as healthy or very healthy, suggesting that public health messages intended for rural housewives need to be more tailored to health-related consequences of fatness.

## Introduction

The world has experienced enormous health improvement in the last century, particularly in its later half (1950’s to 2000). Despite the overall improvement, however, we also have to acknowledge that developing countries benefited unequally from the above health gains, with many countries continue to have high mortality rate, where in some parts of the world the burden of ill health in the form of infectious and parasitic diseases are still prevalent. Communicable disease is an avoidable disease and avoidable mortality, but due to unequal access to healthcare and preventive remedies within a country can lead to notable number of death as a result of lack of access to effective treatment [1].

**Figure 1:**
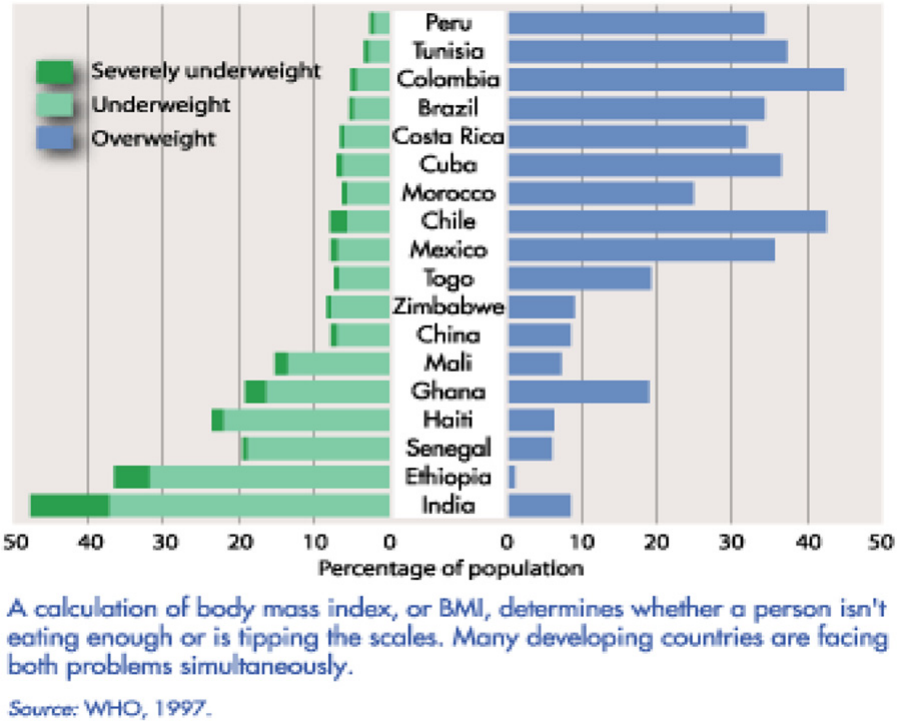
**Underweight and overweight in selected developing countries.**

Developing countries particularly those in the middle range of GNP are currently facing a double burden of malnutrition at both extreme end of the same continuum, undernutrition and obesity [2]. Both undernutrition and obesity have wide ranging health consequences in all age groups. Figure 1 show a few selected developing countries with the double burden of malnutrition. As shown in Figure 1, many countries in Central and Latin America are showing prevalence of overweight above 30% of their population, particularly in Colombia, Chile, Peru, Brazil, Costa Rica, and Cuba. The graph also depicts an increase trend between underweight and overweight in most countries in Latin America and Africa. This problem is not only confining to Latin America or Africa, but is also a common trend in Southeast Asia.

Despite gloomy conditions in terms of global health, the world will at the same time see rapid growth of cities and income in the near future. In 1900 only 10% of the world’s population lived in cities, however, today the proportion has increased to nearly 50% [3]. According to the United Nations estimates, almost all of the world’s population growth between 2000 to 2030 will be concentrated in urban areas of developing countries, where, if the present trend continues, it is expected that 60% of the developing countries will be urban by 2030. At the same time it is projected that income per person in developing countries will grow at an annual rate of 3.4% between 2010-2015, which is twice that, was registered in the 1990’s (1.7%).

Obesity is defined as excess body fat [4]. On the other hand overweight means the body weight is above ideal weight or standard weight for height. A person may be overweight but not necessarily overfat, this is common among athletes or football players [5]. However, normally a person who is grossly overweight will most likely be overfat. The World Health Organization (WHO) defined obesity as those people with the body mass index (BMI) of equal of greater than 30, and overweight as those whose BMI are between 25.0 to 29.9 [6]. At the physiological level obesity can be referred to as a condition of abnormal or excessive fat accumulation in adipose tissue to the extent that health may be impaired [7]. The normal scientific explanation for obesity has been the imbalance between energy intake and energy expenditure. When input is greater than expenditure, excess fat will accumulate. However, understanding the physiological basis alone is not adequate, as it can be seen today that obesity has become a pandemic, there is a trend towards global obesity or globosity [8]. In western countries the prevalence of obesity is beyond control despite the knowledge and research they have accumulated [9,10]. Being obese is associated with increased blood pressure, elevated total cholesterol, abnormal lipoprotein ratios, hyperinsulinemia, and type 2 diabetes [11]. The most prevalent and immediate consequence from obesity, however, may be its negative impact on quality of life [12].

Unfavorable psychological factors, lower self-ratings of health, and worse health-related behavior can be found in overweight and obese individuals. Obese individuals are more likely to be dissatisfied with their body shape and size [13,14]. Weight stigma increases vulnerability to depression, low self-esteem, poor body image, maladaptive eating behaviors and exercise avoidance [15]. Thinness is a beauty ideal in both Europe and the US, so being overweight or obese may contribute to body dissatisfaction and low self-esteem that increases the risk of depression [16]. Some obese people report social anxiety, whereby they are embarrassed to go out because they may not ‘fit’ into a chair in a restaurant or an airplane, for example. Being obese reduces their self-esteem and the effect on their social life leaves them isolated and vulnerable [17].

This study attempted to assess the self-perception and the quality of life among housewives in rural households in the State of Kelantan, Malaysia, and at the same time solicits people’s perception about obesity based on their cultural and socioeconomic context.

## Methods

Population Sample- Respondents of this study were selected by cluster sampling from a list of rural villages within a sub-district that were selected by random sampling from 8 subdistricts in the District of Bachok in the State of Kelantan, Malaysia.

Included in the study were female housewives aged 20 years and over, with body mass index above 25. Other inclusion criteria were being healthy and not suffering from any serious diseases, Non-pregnant and giving written consent to be interviewed and taken body measurements. Excluded were those with ages below 20, body mass index below 25 or suffering from serious illnesses or psychiatric problems. Were also excluded pregnant women and those who did not consent to participate in the study.

The study was approved by The Research Ethical Committee (Human) of Universiti Sains Malaysia (Approval No. USMKK/PPP/JEPeM [207.3.(6)]). The purpose and nature, of the study were explained to all participants, who gave their written informed consent before participation. The study was done in full accordance with the ethical provisions of the World Medical Association Declaration of Helsinki (as amended by the 52nd General Assembly, Edinburgh, Scotland, October 2000).

Sample Size - The sample size for this study was 421 housewives: The primary data was collected using a questionnaire, interview and focus groups methods, where the researcher conducted a field survey among selected groups of respondents in different communities.

The questionnaire focuses on eating habits, body image, quality of life (ORWELL 97) and socio-demography. The focus group discussion touched on globalization of food consumption, lifestyles and socio-cultural perception of obesity.

Quality of Life Assessment - An assessment of quality of life among overweight and obese respondents used the ORWELL97. This questionnaire has been translated into Bahasa Malaysia. Data Analysis - Data entry and analysis was performed using the SPSS for Windows software. The analysis consisted of descriptive and inferential findings to describe the underlying factors and predicting variables in modifying body weight among rural housewives in Malaysia. The result also discussed the quality of life of respondents in relation to overweight and obesity.

## Results

A total of 421 respondents who were all female housewives from 8 sub-districts in the district of Bachok participated in the study (Table 1). The age of respondents were mostly within the range of 20-59 years old with the majority from the 40-59 age group (69.6%), with the mean age of 45.01 + 9.01 (Table 1). In terms of marital status, 86.9% were married and the rest were either widows or divorce (Table 1). Household size and number of children are also shown in Tables 1, with a mean of 6.00 + 2.48 and 5.3 + 3.0 peoples, respectively. More than 64% of the respondents had secondary education, while less than 10% did have any form of formal education (Table 2). As housewives most respondents (66%) did not have personal income (Table 2), while in terms of household income the majority (82.2%) were in the income bracket of below RM1000 per month (Table 2). About 75% of the respondents spent less than RM 500 per month on food for the household, the mean monthly expenses on food was RM 400.62 (Table 2).

**Table 1:** **Socio-demographic data**

**Variables**	**Categories**	**Frequency**	**Percent (%)**
Numbers of sample population and sub-district (n = 421)	Tanjung Pauh	48	11.4
	Tawang	53	12.6
	Perupok	53	12.6
	Melawi	47	11.2
	Bekelam	54	12.8
	Gunong	50	11.9
	Mahligai	65	15.4
	Telong	51	12.1
**Age group of respondents** (n = 421) Mean age = 45.01 ± 9.01 years old	20 – 29	23	5.4
	30 – 39	96	22.8
	40 – 49	152	36.1
	50 – 59	141	33.5
	60 and above	9	2.1
**Marital status** (n = 421)	Married	366	366
	Divorce	10	10
	Widow	45	45
**Size of households** (n = 421) Mean size of households: 6.00 ± 2.48 peoples	1 – 4	151	35.9
	5 – 9	238	56.5
	>9	32	7.6
**Numbers of children living in households** (n = 421) Mean numbers of children living in households: 5.3 ± 3.0 peoples	None	18	4.3
	1 – 4	154	36.6
	5 – 9	207	49.2
	10 – 14	39	9.3
	15 and above	3	0.7

**Table 2:** **Socio-economic data**

**Variables**	**Categories**	**Frequency**	**Percent (%)**
**Education level of respondents** (n = 421)	No schooling	38	9.0
	Primary school	111	26.4
	Secondary school	271	64.4
	Higher education	1	0.2
**Personal income of respondents** (n = 421)	No income	278	66.0
	Below RM 499	105	24.9
	RM500 – RM999	34	8.1
Mean personal income: RM118.8 ± 206.0	RM1000 and above	4	1.0
	No income	278	66.0
**Household income of respondents** (n = 421)	Below RM499	132	31.4
	RM500 – RM999	214	50.8
	RM1000 – RM1499	43	10.2
Mean household income; RM683.71 ± 456.4	RM1500 – RM1999	16	3.8
	RM2000 and above	16	3.8
**Food expense (RM)**	Below RM 499	315	74.8
per month by respondents (n = 421) Mean food expenses per month: RM400.6 ± 175.6	RM500 – RM999	98	23.3
	RM1000 and above	8	1.9

The respondents were asked regarding their self-perception of health and physical activities, the findings are shown in Table 3, where 66.7% considered themselves as very healthy or healthy. Almost all of respondents planned to lose weight (96.2%) (Table 3).

**Table 3:** **Self perception on health, weight status of respondents and partners**

**Variables**	**Categories**	**Frequency**	**Percent (%)**
Self perception on health (n = 421)	Very healthy	107	25.4
	Healthy	174	41.3
	Moderately healthy	100	23.8
	Not well	40	9.5
Current weight status of respondents (n = 421)	Planning to lose weight	405	96.2
	Satisfied with current weight	16	3.8
Hierarchy of priority in life of respondents (n = 419)	Physical healthy	229	54.7
	Happy family	124	29.6
	Self-happiness	37	8.8
	Wealthy	14	3.3
	Modest living	10	2.4
	Emotionally healthy	4	1.0
	Higher educational	1	0.2
Current status of spouse’s body weight (n = 366)	Obese	65	17.8
	Overweight	3	0.8
	Normal	243	66.4
	Thin	54	14.8
	Very thin	1	0.3
Expectation on spouse (n = 366)	Lose weight	73	19.9
	Maintain current weight	259	70.8
	Gain weight	34	9.3
	TOTAL	366	100.0
Preferred body weight of sexual partners of respondents (n = 421)	Obese	2	0.5
	Overweight	8	1.9
	Normal	403	95.7
	Thin	8	1.9

The respondents were also asked regarding their priority in life, Table 3 also listed the ranking of priority by respondents. The number one priority in Table 3 is to be physically healthy (54.7%), followed by having a happy family (29.6%), self-happiness, being wealthy, emotionally healthy, modest living, sanity, and earned higher education.

The respondents’ current spouse/partners, expectations and preferred sexual partners in relation to body weight are all shown in Tables 3. More than 66% has spouse or partners who are normal weight and only 18% has obese partner (Table 3). More than 70% of respondents expected their current partners to maintain their current weight and about 20% expected them to lose weight (Table 3). Regarding sexual partners, more than 95% preferred sexual partners who are of normal weight (Table 3).

Tables 4 reported the respondents’ responses on what do an obese and thin person represent. More than 55% said that obesity symbolizes happiness, 19.4% said it reflects sickness, 16.1% thought it was laziness and 5.5% said it was a result of lack of control in food consumption, respectively (Table 4). Regarding thinness, 42.2% thought these people were not happy, 22.7% said it was due to fear of eating, 19.8% thought they may be sick and 9.6% said it reflects a weak person (Table 4).

**Table 4:** **Perception of respondents of a beautiful body, obesity and Satisfaction with current body shape**

**Variables**	**Categories**	**Frequency**	**Percent (%)**
Obesity symbolizes by respondents (n = 330)	Rich/affluent	6	1.8
	Strong	7	2.1
	Happy	182	55.2
	Lack of control in food consumption	18	5.5
	Laziness	53	16.1
	Sickness	64	19.4
Thinness symbolizes by respondents (n = 313)	Poor	10	3.2
	Weak	30	9.6
	Unhappiness	132	42.2
	Fear of eating	71	22.7
	Laziness	7	2.2
	Sickness	62	19.8
	Others	1	0.3
Defining beautiful women by respondents (n = 421)	Facial attractiveness	132	31.4
	Shape of the body	102	24.2
	Hair style	1	0.2
	Voice	2	0.5
**(Features)**	Behavior	184	43.7
Defining a handsome man by respondents (n = 421)	Facial attractiveness	98	23.3
	Shape of the body	110	26.1
	Hair style	1	0.2
**(Features)**	Behavior	212	50.4
Perception of respondents of a beautiful/body (women)(n = 421)	Fat	19	4.5
	Muscular	2	0.5
	Tall	174	41.3
	Short	1	0.2
**(Features)**	Thin	225	53.4
Satisfaction with their current body shape (n = 421)	Yes	40	9.5
	No	381	90.5

The perception in defining what a beautiful female person is presented in Table 4. Most respondents rated behavior and personality (43.7%) as the most important indicator, followed by facial (31.4%) beauty and the shape of the body (24.2%). In defining a handsome male, behavior and personality also was rated highest (50.4%), followed by body shape (26.1%) and facial attractiveness (23.3%) (Table 4). Table 4 also represents the perception of respondents with respect to a beautiful body or shape. For female, thin or slenderness was considered as the most important attribute (53.4%), followed by height (41.3%) (Table 4). While for males, a beautiful body can be defined as being tall (67.9%), followed by thin (17.6%) and being muscular (10.5%) (Table 4).

On body self-perception, 90.5% are not satisfied with their current body shape (Table 4), the main reason why they are not satisfied is because they perceived they are obese or overweight.

A self-reported measure of obesity –related quality of life questionnaire (ORWELL 97) was administered to the respondents to assess whether their weight affect their quality of life [18]. ORWELL 97 consisted of an 18 item questions and for each item the respondent scored on a 4-point Likert scale the occurrence and severity of the symptom (occurrence) and the subjective relevance of the symptom-related impairment in the respondent’s own life (relevance). The score of the item is calculated as the product of occurrence and relevance. The total ORWELL 97 score is obtained as the sum of the scores of individual items. Higher ORWELL 97 scores mean a lower quality of life.

The results of ORWELL 97 scores for the entire data are shown in Table 5, with the mean total score of 47.7 ± 35.2. The mean ORWELL 97-O (occurrence) is 25.3 ± 16.3, and the mean ORWELL 97-R (relevance) is 22.4 ± 18.9.

**Table 5:** **ORWELL 97 total score for all respondents (n = 421)**

**Questionnaires**	**R+ O**	**R**	**O**
1.	4.27 ± 2.05	2.69 ± 0.76	1.58 ± 1.29
2.	2.69 ± 2.44	1.88 ± 1.21	0.81 ± 1.23
3.	1.20 ± 1.85	0.65 ± 0.94	0.55 ± 0.91
4.	2.41 ± 2.02	1.02 ± 1.02	1.39 ± 1.00
5.	2.06 ± 2.13	0.76 ± 0.94	1.30 ± 1.19
6.	4.44 ± 1.81	2.36 ± 0.90	2.08 ± 0.91
7.	4.46 ± 1.35	2.98 ± 0.20	1.48 ± 1.15
8.	4.56 ± 1.70	2.94 ± 0.39	1.62 ± 1.31
9.	2.41 ± 1.97	1.79 ± 1.14	0.62 ± 0.83
10.	3.36 ± 1.90	0.87 ± 1.02	2.49 ± 0.88
11.	2.23 ± 2.49	0.95 ± 1.21	1.28 ± 1.28
12.	1.08 ± 1.68	0.30 ± 0.76	0.78 ± 0.92
13.	1.86 ± 2.06	0.76 ± 0.97	1.10 ± 1.09
14.	1.86 ± 1.91	1.14 ± 0.96	0.72 ± 0.95
15.	1.36 ± 1.75	0.86 ± 0.97	0.50 ± 0.78
16.	1.50 ± 1.84	0.79 ± 0.90	0.71 ± 0.94
17.	2.85 ± 1.93	0.80 ± 0.90	2.05 ± 1.03
18.	3.06 ± 2.27	1.74 ± 1.10	1.32 ± 1.17
**Total**	**47.7 ± 35.2**	**25.3 ± 16.3**	**22.4 ± 18.9**

## Discussion

Understanding community views and perceptions in regards to health and obesity is essential to design and achieve successful health promotion strategies. The actions people take to maintain their health depend on how they perceive the threat of the disease. In other words, when people perceive that they are susceptible to a disease and are likely to suffer serious consequences from it, then they tend to take action to prevent it. This study aimed to explore community perception of obesity and obesity related quality of life among overweight and obese housewives in rural areas in Bachok District, Kelantan, Malaysia. The results of the survey show a common trend regarding the perception of people in relation to health, dietary practices and obesity. Even though more than 66 percent of the respondents perceived themselves as healthy or very healthy, 96.2% said they plan to lose weight, which means that although they are overweight still some of them considered themselves as healthy. This result was unexpected as overweight and obese respondents are more likely to report poorer health in comparison to those with normal weight [19], given that studies have demonstrated that there is no healthy pattern of increased weight [20]. The high percentage of obese and overweight rural housewives in Bachok on higher self-reported health status could be explained by their low socioeconomic status. Indeed, a negative association between high education and poor self-reported health was found in a recent study implying women in St. Petersburg, Estonia and Finland [19]. In St. Petersburg unlike the other two areas, housewives rather than employed women had less often poor perceived health. Housewives in Bachok had low socioeconomic status, and most of them had personal and household income below the current minimum basic wages of RM900 in Peninsular Malaysia, as well as education level below higher education. A quarter of the respondents had spouses who were overweight or obese. Thus considering the respondent’s population are already a group of overweight people, about two third of them have spouses who have normal weight.

The results of body self-perception was expected, because the respondents that we selected were mostly overweight or obese (Mean BMI = 32.1) (result under publication elsewhere). It is interesting to also note that even though a whopping 90.5% of the women were not satisfied with their body shape, a high percentage of respondents perceived that obesity symbolizes as being happy, which seemingly reflect that it’s alright to be obese and only happy people have good appetite. Likewise, thinness symbolized people who are not happy and those who feared or resisted eating, thus avoid eating or lacking in appetite. They are also being perceived as sick and weak. Happiness here is perceived as an obesogenic factor as it is tied to comfort eating and weight gain. This finding corroborates recent study [21] which reported that happier people are more likely to overeat compared to unhappy individuals. On the other hand, a substantial proportion perceived obese people as those who are sick and lazy, people can be sick as a result of imbalances in body metabolism or an indulgence in consumption of food. Lack self-control is also seen as one the characteristics of obese people, lack of control here can mean inability to resist food and eating temptation or people who lack overall self-discipline. In terms of placing their priority in life, the greatest proportion chose physical health as the number one priority, having a happy family is the second priority. The third priority is self-happiness or self-contented, and the fourth placing is being rich. This results show the close relationship between being healthy and having a happy family, including personal happiness.

The results of the perception of beauty show how important is the character or behavior of a person in society, and it has a very powerful influence in determining the acceptability by the society at large. This may be unique to Malaysia where a person’s worth is in his/or her behavior, you are evaluated on how you conduct yourself within a certain norms that is expected in your society. This is also not surprising because the housewives are from one of the most culturally conservative and prudish States in Malaysia where attractiveness and person’s worth are socially based on character rather than body shape and facial look as in the western societies. Nevertheless, when it comes to their perceptions of the ideal body size, respondent preferences were highest for the thin figure. This paradox could be linked to the nutritional and cultural transition that is accompanying the globalization and rapid growth of the Malaysian economy with the concomitant acculturation into western societies. Thinness is indisputably a striving for the beauty ideal in modern western societies because of the socially constructed idea that physically attractiveness is one of the women’s most important assets. This study suggests that the values associated with self perception on health, thinness and obesity could be influenced by socio-cultural conditions.

The evaluation of the relationship between obesity and quality of life is not always a direct relationship because of the various domains or components of the quality of life measures. For this study the obesity related well-being (ORWELL 97) was used as an instrument for the assessment of the quality of life of respondents [12,18]. Past studies have reported that obese individuals had a poorer physical quality of life than normal individuals [22,23], this condition is also related to the impaired physical well-being among obese individuals. Thus the impact of weight on physical and psychological well-being is a very important area that need further research. The results of the ORWELL 97 of the total score are comparable to the mean total score of the population studied by Mannucci in Italy (1999), which is 47.9. However, the scores for both ORWELL 97 – O and ORWELL97 – R, were lower than the Italian population. According to the interpretation of ORWELL scores a lower scores mean a better quality of life. This results also differed from the total ORWELL 97 findings from Indonesia (57.71 ± 37.60), Philippines (52.61 ± 32.99), and Thailand (50.98 ± 32.14) [24], which may mean that overweight and obese respondents in Bachok have a better quality of life than their counterparts in Thailand, Philippines, and Indonesia.

## Conclusion

This study surveyed the perception of rural housewives population regarding health, obesity and impact of weight on quality of life. The results indicated that perception on obesity did not differed very much between respondents, in fact there existed a lot of similarities in their perception about health, quality of life, personal health and self-satisfaction with own body. However, their quality of life was within the normal or moderate level based on the ORWELL 97 assessment. Even though most of the respondents were aware of their body weight and indicated an intention to lose weight they also reported themselves as healthy or very healthy, suggesting that public health messages intended for rural housewives need to be tailored to health-related consequences of fatness.

This study is a preliminary study, and the results of the study is very encouraging, it challenged the researchers to go into more in depth to untangle the link between nutrition and socio-cultural behaviors and health consequences, particularly obesity. It is hoped that further research can be carried out to provide a more comprehensive findings regarding the factors and variables that are at play in accelerating or slowing down dietary consumption and physical activities.
